# Deciphering key factors in pathogen-suppressive microbiome assembly in the rhizosphere

**DOI:** 10.3389/fpls.2023.1301698

**Published:** 2023-12-05

**Authors:** Yohannes Ebabuye Andargie, GyuDae Lee, Minsoo Jeong, Setu Bazie Tagele, Jae-Ho Shin

**Affiliations:** ^1^ Department of Applied Biosciences, Kyungpook National University, Daegu, Republic of Korea; ^2^ Department of Plant Sciences, Bahir Dar University, Bahir Dar, Ethiopia; ^3^ Department of Microbiology and Plant Pathology, University of California, Riverside, Riverside, CA, United States; ^4^ Department of Integrative Biology, Kyungpook National University, Daegu, Republic of Korea; ^5^ Next Generation Sequencing (NGS) Core Facility, Kyungpook National University, Daegu, Republic of Korea

**Keywords:** domestication, homeostasis, soil-borne pathogens, soil nutrients, symbiosis

## Abstract

In a plant-microbe symbiosis, the host plant plays a key role in promoting the association of beneficial microbes and maintaining microbiome homeostasis through microbe-associated molecular patterns (MAMPs). The associated microbes provide an additional layer of protection for plant immunity and help in nutrient acquisition. Despite identical MAMPs in pathogens and commensals, the plant distinguishes between them and promotes the enrichment of beneficial ones while defending against the pathogens. The rhizosphere is a narrow zone of soil surrounding living plant roots. Hence, various biotic and abiotic factors are involved in shaping the rhizosphere microbiome responsible for pathogen suppression. Efforts have been devoted to modifying the composition and structure of the rhizosphere microbiome. Nevertheless, systemic manipulation of the rhizosphere microbiome has been challenging, and predicting the resultant microbiome structure after an introduced change is difficult. This is due to the involvement of various factors that determine microbiome assembly and result in an increased complexity of microbial networks. Thus, a comprehensive analysis of critical factors that influence microbiome assembly in the rhizosphere will enable scientists to design intervention techniques to reshape the rhizosphere microbiome structure and functions systematically. In this review, we give highlights on fundamental concepts in soil suppressiveness and concisely explore studies on how plants monitor microbiome assembly and homeostasis. We then emphasize key factors that govern pathogen-suppressive microbiome assembly. We discuss how pathogen infection enhances plant immunity by employing a *cry-for-help* strategy and examine how domestication wipes out defensive genes in plants experiencing *domestication syndrome.* Additionally, we provide insights into how nutrient availability and pH determine pathogen suppression in the rhizosphere. We finally highlight up-to-date endeavors in rhizosphere microbiome manipulation to gain valuable insights into potential strategies by which microbiome structure could be reshaped to promote pathogen-suppressive soil development.

## 1 Introduction

Soil-borne plant pathogens pose a complex and sustainable challenge to crop production. The soil serves as a nurturing environment for phytopathogens that cause substantial production losses. It also shelters beneficial microbes that offer imminent solutions by combating important soil-borne pathogens such as *Fusarium* (Zhang et al., 2022; Sapkota et al., 2023), *Verticillium* (Lazcano et al., 2021; Zhao et al., 2021b), *Ralstonia* (Wang et al., 2019; Yang et al., 2023), *Rhizoctonia* (Carrión et al., 2019) and nematodes (Silva et al., 2022).

Soil suppressiveness to phytopathogens is mainly attributed to the activity of soil-residing microorganisms (Carrión et al., 2019). According to Baker and Cook (1974), pathogen-suppressive soil is defined as the “*inhospitality of certain soils to some plant pathogens where either the pathogen cannot establish, they establish but fail to produce disease, or they establish and cause disease but diminish with the continued culture of the crop.*” Pathogen suppression in soils is further classified into two major categories, *general suppression*, and *specific suppression* (Weller et al., 2002). *General suppression* is caused by an integrated collaboration of all microbes residing in the soil. It is effective against a wide range of soil-borne pathogens, but its effect cannot be transferred to conducive soils (Weller et al., 2002; Cook, 2014). It is generally regarded as a cumulative natural soil attribute that cannot be deteriorated or lost in the absence of a plant (Corke, 1975). *Specific suppression* is the result of a single microbe or a community of microbes targeting a single pathogen or pathogens in the same genus (Corke, 1975; Weller et al., 2022). *Specific suppression* can be transferred to conducive soils when inoculated with 0.1%–10% or less (w/w) suppressive soil (Weller et al., 2002; Cook, 2014; Weller et al., 2022).

The rhizosphere is a narrow zone of soil surrounding living plant roots where the residing microbes and their activities are influenced by the plant through root exudates (Curl and Truelove, 1986). It is a principal site for complex plant-microbe interactions, and it may also serve as a platform for interspecific plant interactions (Zhou et al., 2023). Hence, the rhizosphere microbiome population structure, composition, and function offer valuable knowledge of the underlying interactions between plants and their associated microorganisms that cause pathogen suppression. Scientists argue whether changes in the microbiome structures are causal factors leading to diseases (Li et al., 2023a) or if they are merely consequences of diseases (Kuang et al., 2023). Yet more investigations are needed to determine this phenomenon, numerous studies have demonstrated that the enhanced pathogen suppression in the rhizosphere is the result of changes in microbiome composition (Carrión et al., 2019; Chen et al., 2020a; Chen et al., 2022; Hong et al., 2023; Khatri et al., 2023b; Wang et al., 2023c; Zhu et al., 2023). Nevertheless, limited efforts have been devoted to systematic manipulation of the rhizosphere microbiome however, more insights have been gained through simplification of the core microbiome and developing synthetic microbial communities (Hong et al., 2023; Zheng et al., 2023).

Biomolecular networks between the host plant and the associated microbes are determinants in microbiome functioning; a comprehensive analysis of various factors involved in the system is crucial. From the ‘*holobiont*’ perspective, the intricate microbial interactions become more complex due to the combined effects of biotic and abiotic factors. So, it is challenging to predict the resulting microbiome structure and function when a certain factor undergoes alterations. The application of high-throughput sequencing techniques and metagenomic tools is instrumental in studying structural and functional dynamics; however, the influence of other factors could result in poor estimation of the microbiome structure and functions if their effect is not properly estimated. Moreover, a substantial portion of the soil microbes are not culturable (Youseif et al., 2021); hence, rhizosphere microbiome manipulation may also rely on deploying indirect monitoring strategies of microbes using factors that govern their assembly.

Numerous factors that influence pathogen suppression in the rhizosphere through microbiome modifications have been identified so far in various studies. While each factor may offer unique benefits, it is crucial to recognize that the rhizosphere system is intricate and interconnected. Hence, modifying a single factor may not result in a substantial benefit in pathogen suppression, and alterations in other factors could potentially reverse the microbiome composition and structure. This may consequentially result in the loss of gained microbial functions. This highlights the need for a comprehensive understanding and analysis of additional factors to achieve consistent and predictable results.

In this context, we consolidate various factors that influence pathogen-suppressive rhizosphere microbiome assembly through comprehensive explorations of current research. Thus, we point out crucial factors that play key roles in pathogen-suppressive microbiome assembly. We give particular emphasis on pathogen infection, domestication, soil pH, and nutrient availability. We also provide a comprehensive overview of their role in rhizosphere microbiome modification and highlight strategies by which successful microbiome manipulations for pathogen-suppressive soil development could be attained. With our exploration, we aim to gain valuable insights into potential inputs for shaping the rhizosphere microbiome structure and functions systematically. This will substantially contribute to future research efforts targeting pathogen-suppressive soil development.

## 2 Soil suppressiveness to phytopathogens in the rhizosphere

The rhizosphere microbiome population provides an additional layer of protection to the plant’s immune system, which is termed the *first line of defense* (Weller et al., 2002; Carrión et al., 2019). In a broad view, rhizosphere microbes can provide protection in two ways; directly by confronting pathogens (Woo et al., 2023), or indirectly by inducing the host to protect against pathogens through various mechanisms (Carrión et al., 2019; Liu et al., 2021).

The cumulative effect of complex interactions among microbes inhibited in the soil helps determine whether the soil promotes disease suppression or fosters disease development (Weller et al., 2022). A wide range of microbial communities are involved in soil suppressiveness. The bacterial communities usually receive greater attention regarding soil suppressiveness (Carrión et al., 2019; Khatri et al., 2023b). The fungal communities are also important contributors to pathogen suppression (Khatri et al., 2023a). Moreover, recent studies have revealed the detrimental effects of phage communities on soil suppressiveness (Wang et al., 2019; Pratama et al., 2020; Yang et al., 2023). The presence and absence of root-knot nematodes in the rhizosphere have also revealed considerable differences in fungal and bacterial community diversities (Silva et al., 2022). Additionally, synergistic damage inflicted by nematode–fungus combinations has been associated with shifts in rhizosphere microbiome composition (Back et al., 2002). Other microbes such as oomycetes and archaea, including arbuscular mycorrhizal fungi and other biotrophic entities in the rhizosphere assist in suppressive soil development (Philippot et al., 2013). Hence, broader microbial interactions shall be considered in suppressive soil development.

To promote soil suppressiveness, beneficial microbes in the rhizosphere can be enriched by promoting their assembly (Chen et al., 2020a; Leite et al., 2023; Wang et al., 2023c; Wantulla et al., 2023) or transferring pathogen-suppressive microbes into conducive soils (Zhao et al., 2021b; Shao et al., 2022; Sritongon et al., 2023). These enrichments have shown a great potential for improving plant health through minimized disease pressure. Furthermore, beneficial microbes play an early warning role, triggering plants to recruit beneficial microbes by using a “*cry-for-help*” strategy when they are provoked by phytopathogens (Carrión et al., 2019).

## 3 Rhizosphere microbiome assembly and homeostasis

The relative abundance, community structure, and network of the microbiome vary in different parts of plants (Zhang et al., 2022; Sapkota et al., 2023). However, the most abundant microbial assembly is found in the rhizosphere (Wang et al., 2022b) as plants expense up to 40% of plant-fixed carbon through root exudates to promote microbial assembly (Weller et al., 2022).

Plants use pattern recognition receptors (PRRs) to detect microbial molecular features such as MAMPs. Then, plants distinguish between beneficial and pathogenic microbes though they both have the same MAMPs (Zhou and Zhang, 2020), and allow association with beneficial microbes while eliciting immune responses to pathogenic ones (Teixeira et al., 2019). Efforts have been made to explain the underlying mechanism in how plants distinguish between commensals and pathogens, yet it is not fully understood. Since commensals do not cause cell damage, their association could be promoted due to the absence of multiple PRR expressions (Zhou and Zhang, 2020). It may also be due to the ability of some commensal bacteria that undergo an immunoregulatory activity to suppress MAMP-triggered immunity (Yu et al., 2019; Teixeira et al., 2021). Otherwise, according to Colaianni et al. (2021), root colonization by commensals could be attributed to non-immunogenic Flg22 epitope variants, which can evade defense activation. Given various attempts made, further investigations are inevitable to fully understand how plants maintain homeostasis in the rhizosphere.

The associated microbes help plants acquire nutrients and provide protection against phytopathogens (Bulgarelli et al., 2013; Trivedi et al., 2020), and the plants in turn provide protection for the microbial community by maintaining the equilibrium through MAMPs. Any disruption to this equilibrium can potentially cause improper microbial assembly that may lead to dysbiosis and causes negative health effects on plants (Chen et al., 2020b; Lee et al., 2021; Wang et al., 2022b). As a result, the plant regulates the system and maintains homeostasis in a mechanism that is not well understood yet (Zhou and Zhang, 2020). Holistically, the “*Holobiont*,” which includes biomolecular networks among the host and the entire microbiome associated with it (Bordenstein and Theis, 2015) has been considered a major contributor to the system’s overall stability (Trivedi et al., 2020).

## 4 Factors determining pathogen suppressive microbiome assembly

Several biotic and abiotic factors are involved in rhizosphere microbiome assembly in the rhizosphere, but pathogen infection, domestication, soil pH, and nutrient availability are closely correlated with pathogen suppression. Understanding the fundamental processes through which these factors influence the microbiome structure and functions is essential for monitoring and manipulating the factors systematically.

### 4.1 Pathogen infection enhances soil suppressiveness

**Figure 1:**
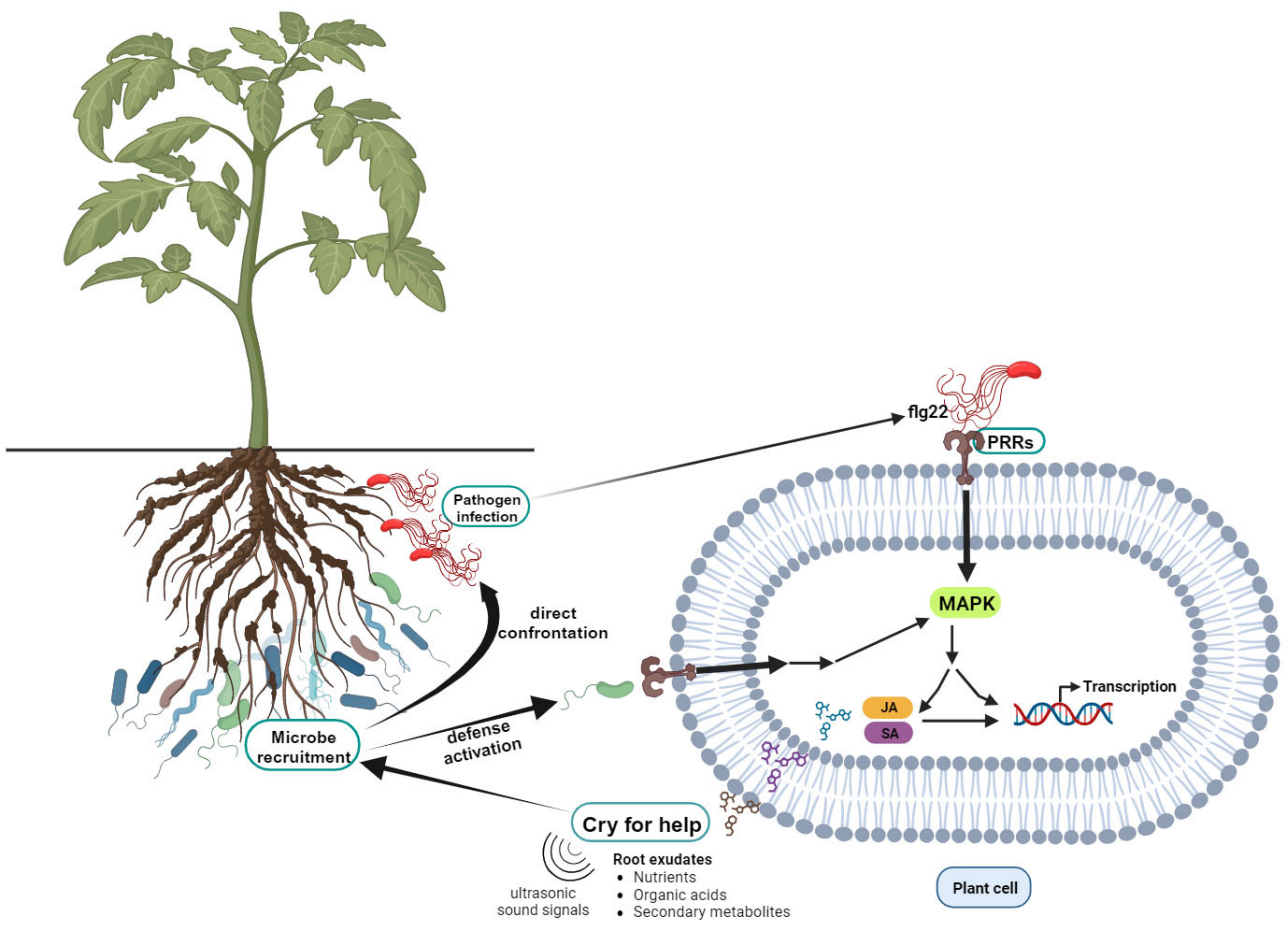
Illustration of pathogen infection initiating defensive microbiome recruitment through the *cry-for-help* strategy. When cell surface receptors such as pattern recognition receptors (PRR) detect the microbe associated molecular patterns of pathogens (Example: flg22 in bacteria flagellin) the cell releases chemical signals and nutrients seeking help from surrounding microbes. Microbes recruited in such a way confront pathogens directly or trigger the plant to activate defense systems such as Jasmonic acid (JA) and Salicylic acid (SA) pathways.

Under normal conditions, plants recruit microbes for their proper functioning in the ecosystem. However, they specifically select and enrich certain microbes when they are under attack by pathogens (Yuan et al., 2018; Carrión et al., 2019; Li et al., 2022). Up on infection by phytopathogens, plants use cell surface and intracellular immune receptors and detect immune signals from pathogens (Zhou and Zhang, 2020). As a result, they release various signals to the surrounding environment which is termed a *‘cry-for-help’* strategy, and eventually, they respond by activating their defense system (**Figure 1**). The main mode of communication involved chemical signals released through the root exudates (Wen et al., 2023); however, a new breakthrough revealed that ultrasonic sound signals could also be employed as part of the *‘cry-for-help’* strategy (Waqas et al., 2023).

Studies have revealed the characteristics of suppressive soils, specifically the underlying mechanism of *“take-all decline”* (Cook et al., 1995); and they discovered the disease decline is the result of systemic acquired resistance gained through defensive microbe recruitment (Liu et al., 2021). The phenomenon has not been considered from this paradigm in the beginning; however, current studies have indicated pathogen-induced microbiome recruitment and its remarkable effect on defensive microbiome assembly (Carrión et al., 2019; Kuang et al., 2023). The recruited microbes will induce systematic resistance in plants, while they may also directly confront pathogens in the system (**Figure 1**) through the secretion of secondary metabolites or competition for nutrients and space (Getzke et al., 2023).

#### 4.1.1 Microbe-pathogen direct confrontation

The plant gains advantages from the recruited microbes, as these microbes engage in direct combat with pathogens through different mechanisms. One of the mechanisms is competing with pathogens for nutrients. Competition for siderophore-mediated interactions in *Bacillus*, *Enterobacter*, and *Chryseobacterium* suppress pathogens and prevent infection by depriving them of access to iron by producing siderophores that the pathogens cannot use (Gu et al., 2020). Some microbes may compete for colonizing the roots as in a *Pseudomonas fluorescens* strain that defends a plant against pathogens without direct antagonism (Wang et al., 2022a). The other mechanism of direct confrontation is through the production of a diverse spectrum of secondary metabolites and enzymes. Antibiotics in bacteria and mycotoxins in fungal antagonists are commonly used by beneficial microbes to inhibit the growth of pathogens in the rhizosphere (Dimopoulou et al., 2021; Clough et al., 2022; Zaid et al., 2022).

#### 4.1.2 Microbiome-mediated systemic protection

The other important mechanism that recruited microbes employ to defend plants against pathogen suppression is indirectly by triggering plants to initiate their defense system against the pathogen (Liu et al., 2021). Biopriming seeds with bioagents activated systemic resistance against seed-borne pathogens by expressing defense-related genes and lignifying the cell wall (Yadav et al., 2023). Beneficial microbes can also systematically induce root exudation of metabolites (Korenblum et al., 2020) which will in turn serve as a protection against pathogens. Such plant-microbe communication may extend further to the neighboring plants (Vlot et al., 2021), and the inter-plant crosstalk can ultimately enhance pathogen defense across the surrounding environment.

Beneficial microbes recruited in this manner may ultimately become part of the core microbiome, and this trait can be inherited by subsequent generations (Toju et al., 2018) through ongoing crop cultivation in pathogen-rich environments. As a result, suppressive soils capable of inhibiting pathogens can be established. In this context, plants preferably recruit a functional core microbiota instead of a taxonomic core microbiota, depending on a specific genotype and environment (Lemanceau et al., 2017; Richardson et al., 2021). Therefore, pathogen infection is a key factor that plays a pivotal role in enhancing the plant’s ability to protect itself and establish a suppressive soil through the recruitment of a functional microbiome.

### 4.2 Plant domestication leads to reduced plant fitness for defensive microbe recruitment

In a broad sense, domestication may not necessarily involve human influence in the innate process of natural plant adaptation to a particular niche (Purugganan, 2022). But in a narrow sense, domestication is the selection of plant species from their wild relatives for their desired characteristics and keeping them for continued use (Pickersgill, 2007).

**Figure 2:**
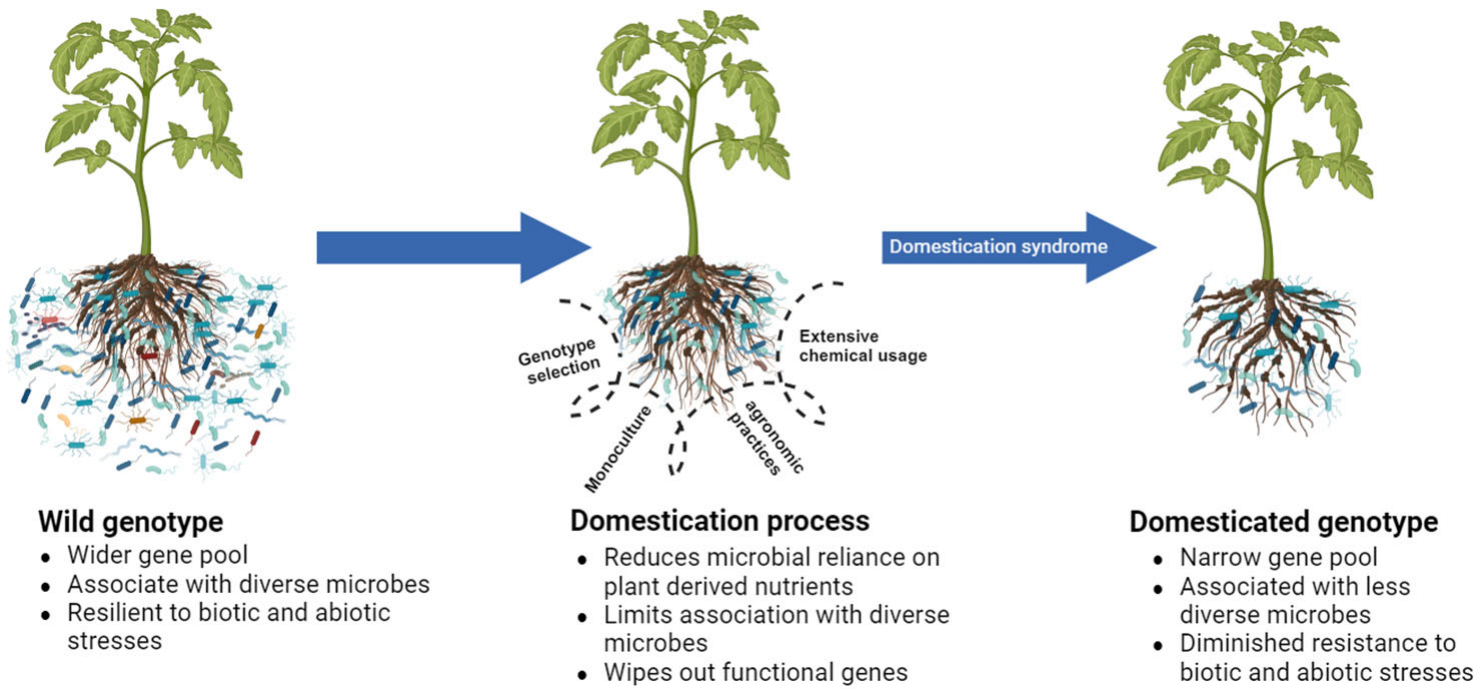
Schematic representation of reduced fitness of domesticated genotypes compared to their wild relatives due to a domestication syndrome they encountered during the domestication process. Plant domestication process wipes out certain functional genes and reduces the association of microbes with plants, ultimately triggering functional losses in plant defense systems.

The selection of plant genotypes based on desired traits is a natural means by which humans ensure their survival. However, because of the influence of various factors connected to domestication, plants experience *domestication syndrome* (Pickersgill, 2007), leading to reduced fitness compared with that of their wild relatives in different aspects (**Figure 2**). In this context, we explore the factors involved in domestication that contribute to the reduced ability of domesticated plants to establish a beneficial microbial assembly. We also acknowledge beneficial practices in a domestication process that assist plants in retaining beneficial microbes which assist in growth and pathogen defense.

#### 4.2.1 Selection of genotypes for a specific trait

In crop variety development, single or few traits are considered and capitalized while selecting a genotype from its wild relatives. In most cases, these traits are associated with yield (Purugganan and Fuller, 2009; Preece et al., 2017; Izawa, 2022), which in turn wipes out important pathogen-suppressive genes from domesticated germplasms.

Genotypes with pathogen defense traits have a higher efficiency in attracting microbes than susceptible ones (Wen et al., 2023). Likewise, resistant common bean cultivars for *Fusarium oxysporum* have traits that support the higher abundance of specific bacterial families in the rhizosphere (Mendes et al., 2018b). Cultivar resistance has also been investigated merely as a function of its association with beneficial microbial assembly (Mendes et al., 2018a; Lazcano et al., 2021; Wang et al., 2023a). If such traits cannot be considered during selection, the selected genotypes will likely become less efficient in pathogen suppression than their wild relatives do (**Figure 2**).

da Silva et al. (2023) pointed out that the taxonomic similarity of microbes associated with *Phaseolus lunatus* decreased by 57.6% from wild to domesticated genotypes. Such a decreased diversity of associated microbes in domesticated genotypes can trigger the functional loss of a microbial community that contributes to nutrient acquisition and disease defense. Therefore, further studies have been suggested to recover the lost traits during domestication via a “*going-back-to-the-roots*” approach, which involves exploiting microbes from indigenous genotypes (Pérez-Jaramillo et al., 2016).

In this context, resistance breeding using marker-assisted quantitative trait loci may assist in identifying and retaining genes responsible for defensive microbiome recruitment. However, the functional trait trade-offs due to gained resistance shall be considered during the breeding process to ensure that microbe association is not compromised (Nelson et al., 2018; Zuccaro and Langen, 2020). It is also important to fine-tune genes in pathogen-resistant genotypes that are associated with an increased capacity to attract beneficial microbes that assist in pathogen suppression (Liu et al., 2021).

Novel insights have been gained through rhizosphere microbiome-based genome-wide association studies that revealed a heritable association between genotypes and rhizosphere microbiome (Deng et al., 2021; Oyserman et al., 2022). This could enable the prediction of microbial taxa associated with specific genotypes. However, other factors could potentially influence this association as many factors govern microbiome assembly, as genotypes enrich different taxa in varying conditions (Carrión et al., 2019; Hong et al., 2023). This may require further investigations on functional features of the quantitative trait loci and the associated microbes in contrasting conditions which could assist in gaining insights into factor-specific key taxa development.

#### 4.2.2 Monoculture

Domesticated crops are typically cultivated in isolation from other crops to preserve their unique traits and qualities, often as part of a monoculture system. In modern agriculture, because of economic motivations following mechanization and the development of high-yielding varieties and agrochemicals, monoculture has become a prevailing trend (Power and Follett, 1987).

In a mono-cropped agroecosystem, microbiome diversity and function are substantially suppressed (Li et al., 2022), thereby inducing decreased suppressiveness in the rhizosphere. Interestingly, a mono-cropped peanut field faces a yield penalty for the depletion of the overall diversity of microbes in the rhizosphere even in the absence of diseases (Li et al., 2020). Such a loss could have been aggravated by the presence of a pathogen. Li et al. (2023a) also asserted that monoculture is a basic factor influencing the replant disease observed in *Rehmannia glutinosa* by causing a shift in the rhizosphere microbiome structure. Therefore, monoculture plays a crucial role in domestication, considerably influencing the rhizosphere microbiome assembly and ultimately determining soil suppressiveness (**Figure 2**).

#### 4.2.3 Agronomic practices

Agronomic practices include a wide range of activities by which growers improve productivity and plant health. The practices employed, such as preparation, planting techniques, irrigation, nutrient management, weeding, and harvesting are integral components of domestication. Notably, each of these practices exerts a noticeable impact on the assembly of the rhizosphere microbiome, ultimately influencing both structural composition and functional dynamics (Bonanomi et al., 2016; Benitez et al., 2017; Hartman et al., 2018; Legrand et al., 2018; Babin et al., 2019; Gong et al., 2019).

Agronomic practices can influence the assembly of pathogen-suppressive microbiomes in either a positive or negative manner, with the ultimate composition and functioning of the microbiome being determined by the cumulative effects of various practices (Chadfield et al., 2022; Fernandez-Gnecco et al., 2022; Morugán-Coronado et al., 2022). Practices such as intercropping and crop rotation are widely witnessed to promote plant health through modification of rhizosphere microbiome (Neupane et al., 2021; Chadfield et al., 2022; Town et al., 2023; Zhou et al., 2023). On the other hand, monoculture, intensive tillage, and extensive use of chemical fertilizers and pesticides have been reported to deteriorate plant health through modification of microbial diversity in the rhizosphere (Li et al., 2022; Morugán-Coronado et al., 2022; Beaumelle et al., 2023).

Tomato plants in potatoonion and tomato intercropping systems gained systemic resistance to *Verticillium* wilt disease through the recruitment of a pathogen-suppressive microbiome (Zhou et al., 2023). Further examination into the relative abundances of significantly enriched bacterial communities revealed the enrichment of pathogen-suppressive *Bacillus* sp. which can also induce systemic resistance to tomato plants. According to Chadfield et al. (2022), a quantitative assessment of intercropping substantiated a 40% reduction in nematode damage and a 55% decrease in disease incidence for focal crops.

Crop rotation was also found in association with the enrichment of potentially beneficial taxa in a 12-year canola rotation while non-rotated canola was steadily associated with common canola pathogens (Town et al., 2023). Hong et al. (2023) discovered rotation-associated and rotation-unique key taxa from a paper and eggplant rotation that showed a strong antagonistic reaction against banana wilt disease.

While certain agronomic practices offer individual benefits, the cumulative effect of various practices may disfavor the enrichment of beneficial microbes in the rhizosphere. Consequently, domesticated crops may retain a diminished ability to attract beneficial microbes compared with their wild counterparts (da Silva et al., 2023; Luo et al., 2023; Yue et al., 2023).

#### 4.2.4 Extensive use of chemicals

Domestication does not necessarily involve the use of chemical fertilizers and pesticides. However, the utilization of pesticides and chemical fertilizers has remarkably increased since the green revolution, leading to consequential outcomes on the rhizosphere microbial community (Newman et al., 2016; John and Babu, 2021; Sangiorgio et al., 2022), despite substantial achievements in transforming global food production (Pinstrup-Andersen and Hazell, 1985). Regardless of the goals of reducing the effects through optimum utilization (Tilman, 1998), chemical fertilizers and pesticides are being used extensively, and they continue to chronically affect the soil microbiome (Wang et al., 2023b).

Chemical pesticides disrupt the natural equilibrium through their strong effect on the diversity of the soil microbes (Beaumelle et al., 2023) and alter the key microbial taxa, ultimately affecting plant health (Yu et al., 2023). Meanwhile, the application of inorganic nutrients reduces the reliance of the soil microbiome on plant-derived carbon sources (Ai et al., 2015), indicating a potential decrease in the soil–microbiome association with plants (**Figure 2**). (Francioli et al., 2016) showed that long-term fertilization affects the microbial community structure and activity. Hence, a partial replacement of inorganic chemicals with organic amendments (Cai et al., 2017; De Corato et al., 2019; Shi et al., 2023) improves soil suppressiveness and plant health in the rhizosphere. Chemical fertilizers may not necessarily reduce the total microbial abundance in the rhizosphere; however, the effect is the alteration of the microbial composition resulting in functional disruption in the rhizosphere microbiome (Yu et al., 2023). Therefore, the usage of chemical fertilizers and pesticides in domestication is one of the driving factors for changes in microbial community structure and function; it also determines soil suppressiveness to phytopathogens.

### 4.3 Soil pH and nutrient availability determine defensive microbial assembly

**Figure 3:**
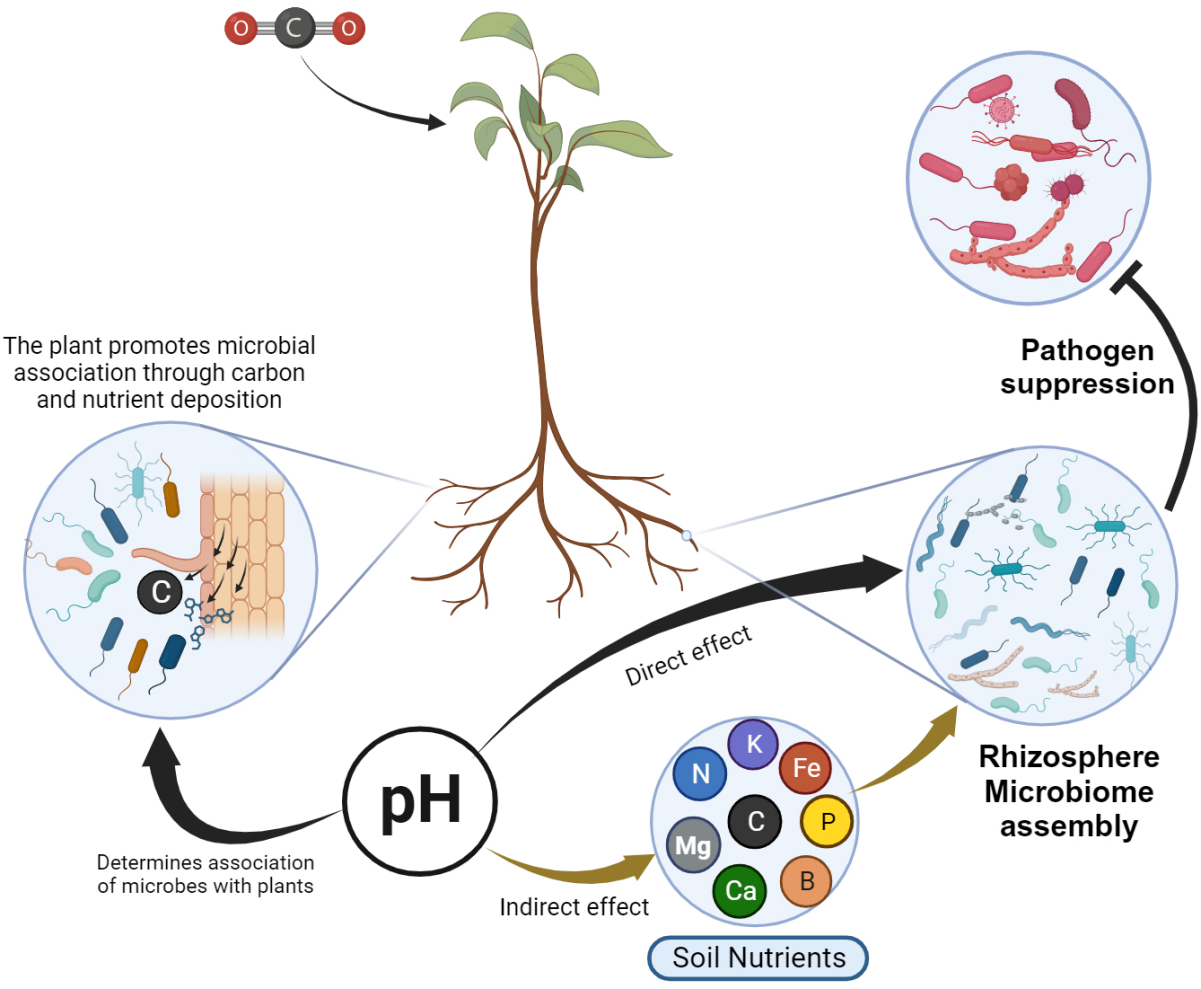
Schematic representation of the direct and indirect influence of pH and nutrient availability in rhizosphere microbiome assembly, and ultimately pathogen suppression. The plant releases its own fixed carbon and nutrients to establish association with microbes. pH has a direct effect on microbial assembly and has an indirect effect through determining nutrient availability.

Symbiotic microbes are biotrophic entities that rely on nutrient availability in the rhizosphere (Spanu and Panstruga, 2017). Hence, to establish a relationship with soil microbes, plants provide nourishment to these microorganisms by expending their own fixed carbon (**Figure 3**), which is released through root exudates (Bais et al., 2006; Weller et al., 2022). The root exudates in turn shape the structure and function of the soil microbial community by monitoring nutrient availability and using other chemical signals (Zhao et al., 2021a). Francioli et al. (2016) indicated that total organic carbon and pH are major factors determining microbial abundance and structure in the rhizosphere, while nitrogen and phosphorus are minor driving factors for microbial assembly in the rhizosphere. As a result, reduced carbon deposition suppresses microbial diversity and function (Li et al., 2022).

Soil pH plays a very important role in shaping the defensive microbial assembly. Consequently, a microbiome from acidified soils demonstrated a diminished ability to defend against pathogen invasion (Li et al., 2023b). As a result, the treatment of acidity in soils enhanced soil health through rhizosphere microbiome modification (Chen et al., 2022). In general, soil pH has a direct effect on microbial assembly and an indirect impact is through determining the element availability (Lammel et al., 2018) that ultimately determines suppressive microbial assembly (**Figure 3**).

Various investigations into organic and conventional systems (Bonanomi et al., 2016; Cavicchioli et al., 2019; Obieze et al., 2023) have revealed variations in microbial composition and diversity. Moreover, studies have highlighted the taxonomic and functional changes attributed to organic nutrient amendments (Cui et al., 2018; Castellano-Hinojosa et al., 2023). Microbial diversity in a Chinese fir (*Cunninghamia lanceolata*) monoculture plantation interacts strongly with dissolved organic matter (Li et al., 2021). Khatri et al. (2023a) also demonstrated that disease-suppressive fungal taxa have been selectively enriched in soils through organic management compared with those enriched through conventional techniques. This can be attributed to the higher organic carbon content (18.43 g kg-1) in the organic soil than in the conventional one (6.5 g kg-1) as carbon deposition is a determinantal factor of microbial assembly.

The ginseng replant disease has been successfully alleviated by the addition of cow dung, which promotes the growth of beneficial microbes, directly by improving nutritional content (Tagele et al., 2023). Interestingly, manure amendment has been observed to enhance the diversity of indigenous bacteria rather than manure-borne bacteria (Schlatter et al., 2022), suggesting that nutrient or organic matter amendment plays a crucial role in fostering microbial diversity (**Figure 3**). However, long-term manure amendments can pose a potential risk of antibiotic-resistant gene contamination through horizontal gene transfer (Yue et al., 2023).

The application of inorganic nutrients positively affects bacterial species diversity in low-nutrient wetlands (Bledsoe et al., 2020). Enebe and Babalola (2020) justified this finding and described the best response of soil microbes for integrated compost and inorganic fertilizer application, which goes in line with similar investigations (Francioli et al., 2016; Kumar et al., 2018). The positive response of soil microbes to inorganic nutrient applications indicates that microbes utilize nutrients regardless of their source (e.g., organic or inorganic sources). The direct influence of nutrient availability on rhizosphere microbiome assembly can be exemplified by the important role of iron in determining soil suppressiveness (Elad and Baker, 1985; Scher, 1986). In iron-deficient environments, microorganisms such as *Pseudomonas* compete for iron by producing siderophores, which in turn suppress the growth of *Fusarium* (Zhu et al., 2023). Furthermore, the nutritional status of plants affects colonization efficiency indirectly, as evidenced by (Hacquard et al., 2016), that plants activate their defense responses under phosphate-sufficient conditions while allowing association under phosphate-deficient conditions. Hence nutrient availability and pH, as demonstrated in various findings, are key factors that determine pathogen defensive microbiome assembly in the rhizosphere.

## 5 Emerging strategies for shaping rhizosphere microbiome

Changes in the rhizosphere microbiome resulted in various effects on plant-pathogen interactions in the underground or aerial niches. Strategies such as soil amendments with nutritive substrates, microbial strain inoculations, and inoculation with synthetic microbial communities have been implemented and brought about considerable results. However, with the advancement of understanding the microbial world, new advances have been made in recent times. In this section, we highlight emerging strategies that could be implemented for shaping the rhizosphere microbiome toward pathogen suppression.

### 5.1 Microbiome-mediated strategies

The rhizosphere is generally colonized by various microbes and the microbiome composition differs greatly between diseased and healthy plants (Kuang et al., 2023). This implies that pathogens can be used to shape the microbiome structure in the rhizosphere. As discussed previously, pathogen-induced microbial assembly is an emerging strategy that results in a distinct microbial assembly that can suppress pathogen infection (Carrión et al., 2019; Kuang et al., 2023). An early warning role discovered in this phenomenon could be used to trigger plant immunity activation that might be evaded or suppressed by pathogens during infection. This suggests the way for the development of suppressive soil development through plant immunization (Oberemok et al., 2022) using pathogens or their derivatives. This could be accomplished by shaping the rhizosphere microbiome so that the defense system can be initiated before real infections occur.

### 5.2 Host-mediated strategies

The strong correlation of pathogen-resistant crop varieties with higher affinity for defensive microbiome recruitment (Wen et al., 2023) casts a light on a new paradigm of resistance breeding, targeting genes responsible for microbiome association rather than just looking for resistance genes in plants (Yue et al., 2023; Zhang et al., 2023). Searching for lost genes responsible for defensive microbial assembly in wild genotypes is an emerging strategy (Oyserman et al., 2022). This will reinforce endeavors for shaping rhizosphere microbiomes *via* engineered plant genotypes that can trigger microbial assembly in the rhizosphere. Host-mediated strategies may also deploy root exudate engineering of the host plant to shape microbial assembly that favors pathogen suppression in the rhizosphere (Yuan et al., 2018; Kawasaki et al., 2021).

### 5.3 Abiotic factor-mediated strategies

Among key factors discussed previously, the agronomic practices, use of chemicals, soil pH and nutrient availability could be managed to shape the rhizosphere microbiome towards pathogen suppression. Through the determination of specific conditions and combinations of factors, a microbiome could be reshaped to suppress specific kinds of pathogens. Emerging insights have been gained to monitor rhizosphere-associated microbes through plant responses to abiotic stresses (Faist et al., 2023). Recent findings revealed that the plant adjusts nutrient availability and rhizosphere pH to monitor microbial recruitment and immune homeostasis (Liu et al., 2023). This suggests that rhizosphere microbiome assembly could be reshaped by nutrient and pH management strategies. Nutrient-driven differences in microbial assembly across different potato landraces also highlighted a great potential in nutrient monitoring for shaping rhizosphere microbiome assembly (Miao and Lankau, 2023).

## 6 Conclusions and future directions

Pathogen suppression is a highly desired trait in the rhizosphere microbiome. Over time, research efforts have been dedicated to enhancing pathogen suppression in the rhizosphere. However, systematic manipulation and accurate prediction of microbiome composition and functions after an introduced change targeting pathogen suppression have been unattainable. The role of biotic and abiotic factors became a prominent challenge as high throughput sequencing and metagenomic tools paved the way for deeper insights into the composition and functional attributes of plant-associated microbes. Remarkably, pathogen infections can lead to a complete shift in the rhizosphere microbial structure, and plant domestication exerts intertwined influences on microbial assembly and structure. Furthermore, soil pH and nutrient availability determine the fate of a microbe in the rhizosphere, that is, whether it will be recruited or rejected. Therefore, future research endeavors toward pathogen-suppressive soil development could be made through microbe-mediated manipulations for plant immunization and defensive microbiome recruitment. Host and microbe-mediated approaches could also be deployed for microbe-assisted crop improvement and microbiome breeding, and using abiotic factor-mediated approaches specific modulation of host-associated microbes could be attainable to improve pathogen suppression in the rhizosphere.

## Author contributions

YA: Conceptualization, Writing – original draft. GL: Writing – review & editing. MJ: Writing – review & editing. ST: Writing – review & editing. JS: Funding acquisition, Supervision, Writing – review & editing.
